# University of Pennsylvania Smell Identification on Iranian Population

**DOI:** 10.5812/ircmj.7926

**Published:** 2014-01-05

**Authors:** Seyed Kamran Kamrava, Mohammad Farhadi, Maryam Jalessi, Babak Khosravian, Behzad Pousti, Ebrahim Amin Tehran, Mohsen Rezaee Hemami

**Affiliations:** 1 Endoscopic Pituitary and Skull Base Unit, ENT-Head and Neck Research Center and Department, Rasool Akram Hospital, Tehran University of Medical Sciences, Tehran, IR Iran; 2 School of Medicine, Tehran University of Medical Sciences, Tehran, IR Iran

**Keywords:** Smell, Odors, University of Pennsylvania Smell Identification Test (UPSIT)

## Abstract

**Background:**

University of Pennsylvania Smell Identification Test (UPSIT) is one of the most common smell identification tests to assess olfactory function.

**Objectives:**

The study was conducted to assess the accuracy of University of Pennsylvania Smell Identification Test (UPSIT) in Iranian population.

**Materials and Methods:**

University of Pennsylvania Smell Identification Test was performed on 30 (50%) female and 30 (50%) male, who were healthy adult volunteers. The total mean score as well as mean scores according to the gender were assessed and compared to the UPSIT classification. Odors considered valid and accurate if its correct identification ability rate was more than 70% among study population.

**Results:**

The test score was 25.04 ± 4.92 in female and 23.29 ± 4.23 in male volunteers that all were considered as microsmia according to UPSIT. Sixteen odorants were correctly identified by about 70% of the volunteers and the remains 24 odorants were identified by less than 70%; 7, 5 and 12 odors was identified by60%-70%, 50%-60% and less than 50% of the volunteers, respectively.

**Conclusions:**

According to the results of the study, documented that even less than half of the odors (16 out of 40) were identified correctly by the volunteers, which indicating that the UPSIT is not a suitable test to evaluate olfactory function in Iranian population due to the high amount of unfamiliar smells that should be replaced with more familiar ones.

## 1. Background

University of Pennsylvania Smell Identification Test (UPSIT) is one of the most common smell identification tests to assess the olfactory function. It has been now translated into several languages and employed widely due to its accurate and appropriate ability to test the olfactory function with no need to complex equipment, and devices (1, 2). However, the identification of different odorants even in a normal population is strongly affected by various social and cultural factors, it is suggested that the test be modified culturally to prevent the cultural biases (2-7).

## 2. Objectives

This study was conducted to assess the accuracy of University of Pennsylvania smell identification test by evaluating the odor identifiability and familiarity in Iranian population.

## 3. Materials and Methods

### 3.1. Study Population

Between April 2006 and April 2011 hundreds of outpatients who referred to our otorhinolaryngology clinic (Iran University of Medical School, Rasool Akram Hospital, Tehran) had a normal otorhinolaryngological examination, 60 healthy volunteers comprising 30 (50%) female and 30 (50%) male (aged 20 - 60 years), enrolled into this cross-sectional study. The ethics committee of ENT- Head and Neck Research Center approved the study, and written informed consent was preoperatively obtained from the patients. The UPSIT was administered to all volunteers and the total mean score as well as the mean scores according to the gender were assessed and compared with the UPSIT classification.

### 3.2. UPSIT Classification

Volunteers considered as anosmia: score of 6 - 18, severe microsmia: score between 19 and 25, moderate microsmia: 26 - 30 in women and 26 - 29 in men, mild microsmia: 31 - 34 in women and 30 - 33 in men and normosmia: score of more than 34 in women and 33 in men.

### 3.3. Identification Ability

The UPSIT has four booklets that each one contains 10 stimuli for smell. The test can be self-administered and uses microencapsulated odorants which are released by scratching the standardized odor-impregnated test booklets. For each stimulus, the respondent chooses out of four options. The ability to identify each odorant was evaluated according to the Doty (8) to determine inaccurate odorants. The cutoff point criterion used by Doty in the validation of each odor was 70% of correct rates (2, 9, 10). Odorant that were identified by less than 70% of the population considered inaccurate as they confounded. Before administration of the test, volunteers also asked to rate their familiarity with each odorant (out of 10) in order to evaluate its correlation with correct answer after applying the test. It was suggested that in the further try to modify the test culturally according to our background, the result could facilitate choosing some new odorant to replace with the unfamiliar ones. 

### 3.4. Statistical Analysis

Data are presented as number (%) , means and standard deviation. Statistical analyses were performed using SPSS 17 for Windows (SPSS Inc., Chicago, Illinois). 

## 4. Results

We analyzed data from 30 (50%) female and 30 (50%) female volunteers, who were tested by using the UPSIT. The test score was 25.04 ± 4.92 in female and 23.29 ± 4.23 in male volunteers. All three scores were considered as severe microsmia according to UPSIT (severe microsmia: score of 19 - 25) and 16 of 40 odors were identified correctly by more than 70% of the volunteers, 24 other odorants were identified correctly by less than 70% of the population and considered as inaccurate odors. 7, 5 and 12 odors were identified by %60-%70, %50-%60 and less than %50 of the volunteers, respectively (Table 1). 

**Table 1.** Correct Identifiability and the Familiarity Level of Odorants ^a^

Odorant	Correct Identifiability	Familiar Level
**Bubble Gum**	91.67 %	7.58 ± 2.11
**Menthol**	80.00 %	7.82 ± 2.39
**Mint**	83.33 %	7.74 ± 2.09
**Banana**	88.33 %	6.94 ± 2.41
**Leather**	75.00 %	5.62 ± 2.83
**Coconut**	83.33 %	7.09 ± 2.51
**Cinnamon**	78.33 %	7.08 ± 2.61
**Ginger bread**	86.67 %	6.44 ± 2.31
**Pine Apple**	80.00 %	7.62 ± 2.19
**Orange**	88.33 %	8.15 ± 1.98
**Watermelon**	76.67 %	7.53 ± 2.16
**Grass**	75.00 %	6.79 ± 2.46
**Smoke**	75.00 %	6.51 ± 2.52
**Soap**	81.67 %	7.25 ± 2.09
**Natural Gas**	88.33 %	6.74 ± 2.76
**Rose**	78.33 %	7.58 ± 2.47
**Onion**	68.33 %	7.62 ± 2.49
**Chocolate**	68.33 %	6.86 ± 2.32
**Lilac**	60.00 %	7.98 ± 1.85
**Peach**	65.00 %	6.51 ± 2.63
**Root Beer**	61.67 %	8.36 ± 2.34
**Pine**	68.33 %	6.36 ± 2.39
**Lime**	68.33 %	7.24 ± 2.32
**Clove**	58.33 %	6.31 ± 2.57
**Liquorice**	55.00 %	4.93 ± 2.81
**Gasoline**	50.00 %	5.24 ± 2.73
**Strawberry**	55.00 %	7.60 ± 2.01
**Peanut**	55.00 %	7.86 ± 2.19
**Pizza**	28.33 %	6.55 ± 2.47
**Cherry**	31.67 %	6.39 ± 2.54
**Motor Oil**	41.67 %	5.01 ± 2.63
**Fruit Punch**	18.33 %	7.67 ± 2.19
**Cheddar Cheese**	31.67 %	3.77 ± 2.61
**Cedar**	21.67 %	6.27 ± 2.38
**Dill Pickle**	23.33 %	5.58 ± 2.57
**Lemon**	31.67 %	6.77 ± 2.60
**Wintergreen**	43.33 %	9.00 ± 1.68
**Thinner**	41.67 %	6.67 ± 2.54
**Grape**	13.33 %	6.75 ± 2.35
**Turpentine**	48.33 %	6.27 ± 2.68

^a^ Data are presented as No. (%) and mean ± SD.

## 5. Discussion

These days, the UPSIT become the most popular olfactory functional test all over the world due to the feasibility to use, the ability to classify primary olfactory dysfunctions or secondary ones caused by neurological diseases with no need to complex instruments (7, 10). It is a 40 item smell identification test comprised of four “scratch-and-sniff” book-lets. It could be administered easily by physicians, nurse practitioners or either could be self-administered. However, despite the worldwide employment of the UPSIT, it is documented that cultural bias has made scientists to adjust the test according to their culture and replace more familiar odors (2-6). We also performed the test in a normal population that revealed the mean score of 25.04 ± 4.92 in female and 23.29 ± 4.23 in male volunteers. According to the classification (severe microsmia: score of 19-25), the scores refer to severe microsmia in both genders in this normal population that showed the inaccuracy of the test in our population. Further analysis revealed that only 16 of 40 odorants were identified correctly by more than 70% of the volunteers including Rose, Natural Gas, Soap, Smoke, Grass, Watermelon, Orange, Pine Apple, Ginger bread, Cinnamon, Coconut, Leather, Banana, Mint, menthol and bubble Gum. It means 24 other odorants with less than 70% correct identifiability are inaccurate and not valid odors based on our cultural background (8, 9). These are 12 odors (onion , chocolate , lilac, peach , root beer, pine , lime ) with %60-%70 identifiability, 5 odors (clove , liquorice , gasoline , strawberry and peanut) with %50-%60 identifiability and 12 odors (pizza , cherry, motor oil , fruit punch, cheddar cheese, cedar , Dill pickle , lemon, winter green, thinner and grape) with less than %50 of the identifiability. These results make us to replace the odorants with less than 70% correct identifiability with some more familiar odors to validate the test in our culture. As our country is a multicultural one, we must identify several odors that could be familiar to most of the races and in all provinces' population, meanwhile have more than 70% correct identifiability. Regarding this matter, we tested extra 15 odors on the participants that showed only 16 out of 40 odors, even less than half of them, were identified correctly by the volunteers that suggest UPSIT is not a suitable test to evaluate olfactory function in Iranian population due to the high amount of unfamiliar smells that should be replaced with more familiar ones.
